# Prevalence of Taurodontism in Premolars and Molars in the South of Iran

**DOI:** 10.5681/joddd.2012.005

**Published:** 2012-03-13

**Authors:** Pegah Bronoosh, Abdolaziz Haghnegahdar, Mehrnoosh Dehbozorgi

**Affiliations:** ^1^Assistant Professor, Department of Oral and Maxillofacial Radiology, School of Dentistry, Shiraz University of Medical Science, Shiraz, Iran; ^2^Private Practice, Shiraz, Iran

**Keywords:** Molar, premolar, prevalence, taurodontism

## Abstract

**Background and aims:**

The study was undertaken to assess the prevalence of taurodontism and related systemic condi-tions and gender differences in premolars and molars of patients attending Shiraz Faculty of Dentistry.

**Materials and methods:**

In this cross-sectional study, panoramic radiographs of 510 randomly selected patients were evaluated by a maxillofacial radiologist for the apically displaced pulp chamber. Detailed medical and family history of the patients was obtained.

**Results:**

The prevalence of taurodontism in 510 panoramic views was 5.5% of patients. Females had significantly higher prevalence of taurodontism than men (P>0.05) and mandibular second molar was the most affected tooth.

**Conclusion:**

Taurodontism was relatively common in population under study. A family history of other anomalies should be checked for affected patients.

## Introduction

Sir Arthur Keith coined the term taurodontism (bull tooth) for the first time in 1913. This term comes from tauros (bull) and odous (tooth).^1^ Taurodontism is a morpho-anatomical variation in the shape of teeth in that the body of the tooth is enlarged and the roots decrease in size.^2^The involved teeth assume a rectangular shape rather than tapering towards the roots. The pulp chamber is extremely large with a greater apico-occlusal height than normal and lacks the usual constriction at the cervical region of the teeth with exceedingly short roots.^3^

Diagnosis of taurodontism has been based on features that are characteristically best visualized on the radiograph.^3^ Although permanent molar teeth are most commonly affected, reports have indicated that taurodontism may not be limited to molars, as it also occurs in the premolar teeth.^4^ Taurodontism may complicate endodontic, orthodontic and/or prosthetic treatment planning.^2^From an endodontist’s view, taurodontism presents a challenge during negotiation, instrumentation and obturation during the root canal therapy. The extraction of a taurodont tooth may be complicated because of a shift in the furcation to the apical third.^2^

The study of pathogenesis of taurodontic root formation revolves around several theories: an unusual developmental pattern, a delay in the calcification of pulp chamber, an odontoblastic deficiency and an alteration in Hertwig’s epithelial root sheath.^5^ Some authors believe that taurodontism is most likely the result of disrupted developmental homeostasis.^6^

Taurodontism has been reported in association with certain syndromes and some genetic defects like hypodontia, Mohr syndrome, Down syndrome, Van der Woude’s syndrome and cleft lip/palate, but its true significance is still obscure.^7 - 10^

Review of the literature reveals a wide discrepancy in the prevalence of taurodontism in different populations. Its incidence has been reported to range between 5.67% and 60% of subjects.^11 - 13^The prevalence of taurodontism in deciduous teeth has been reported to be 0.3%.^14^

Quantitative accounts of prevalence, however, have been limited to studies of different population groups.^15 , 16^ Besides, the majority of studies have not considered both premolar and molar teeth in their prevalence evaluation or have not stated exactly which teeth are assessed.^3, 14,17 - 19^, Few studies have reported the prevalence of taurodontism in Iran;^19^ however, it has not been mentioned which teeth exactly were evaluated. Therefore,to determine a precise prevalence in the Iranian population several studies in different ethnic regions should be carried out. The aim of this study was to assess the frequency of taurodontism in premolars and molars of dental patients in the southern part of Iran by radiographic analysis and to compare the results with published data. In addition, our goal was to determine if any systematic disease is related to this anomaly.

## Materials and Methods

A randomly selected sample of 510 digital panoramic radiographs of patients who had referred to Shiraz Faculty of Dentistry, Department of Oral Radiology, between January and March 2010 were examined. The Cassette used was 15×30 Regius RC-110 (Konica Minolta, Japan). Patients under 14 was excluded based on the apexogenesis time of second molars and if the panoramic radiograph was not of high quality. In addition, carious, restored and fractured teeth, third molars, incomplete apical foramen formation, undetectable furcation and fused roots were not included. All the digital radiographs were viewed in a dark room by one experienced examiner. A sample of 60 radiographs was re-examined by the same examiner approximately four weeks later and an agreement of 100% was obtained.

Diagnostic criteria included visual evaluation of premolar/molar teeth with large pulp chamber in relation to outer tooth configuration, less marked cervical constriction than the normal tooth form, an apically displaced furcation and short roots. Measuring distance (MED.e.COM SARL software, France) between CEJ and the floor of the pulp chamber more than 3 mm was considered as taurodont based on Blumberg index.^20^ Taurodonts were classified as hypotaurodont, mesotaurodont, or hypertaurodont depending on the relative amount of apical displacement of the floor of the pulp chamber.^21^

Finally, patients’ history was reviewed for any possible association with other dental anomalies or co-existing genetic diseases or syndromes.

Statistical analysis of data was performed using SPSS 13 and the frequency distribution for taurodontism was calculated. Chi-squared test was also used to compare the prevalence of taurodontism between male and female subjects (P<0.05).

## Results

The study group comprised 236 (46%) males and 274 (54%) females with a mean age of 27.5 years. The age range was 15‒61 years and the average number of premolar/molars per patient was 14. Of the 7022 premolar/molars examined, 48 (0.68%) teeth were found to have taurodontism. These teeth were detected in 28 (5.5%) of 510 subjects as 11 (39.3%) in males and 17 (60.7%) in females. Females had a higher prevalence of taurodontism than males (61% vs 39%) which was statistically significant (P=0.04) (Table 1).

**Table 1:** Distribution of taurodontism in premolars and molars in the maxilla and mandible by gender

Jaw	Male	Female	Total
Maxilla	8	15	23
Mandible	9	16	25
Total	17	31	48

**Table 2:** Distribution of taurodontism according to morphology by gender

Morphology	Male	Female	Total
Hyper	1	0	1 (2%)
Meso	6	9	15 (31%)
Hypo	10	22	32 (67%)
Total	17	31	48

Analysis of the morphologic index of taurodonts^22^ showed 32 out of 48 taurodonts were of hypotaurodont type (67%), 15 (31%) were mesotaurodont and 1 (2%) was hypertaurodont. The prevalence of hypotaurodonts in male and female was 71% vs 58.8%. However, the difference was not statistically significant (P=0.332) (Table 2).

A cluster analysis of total taurodonts in the mandible (52%) versus maxilla (48%) of both males and females combined showed a statistically significant difference (P<0.05). Table 3 demonstrates distribution of taurodonts. Mandibular second molar was the most common tooth involved in the female, followed by maxillary second molar; however, in males the distribution was nearly equal.

**Table 3:** Distribution of taurodontism according to affected teeth

Tooth	No
Mandibular 2nd molar	19
Maxillary 2nd molar	13
Maxillary 1st molar	8
Mandibular 1st molar	3
Mandibular 2nd premolar	3
Maxillary 1st premolar	2
Total	48

Out of 28 patients with taurodont teeth, ten had a history of systemic disease or other abnormalities, such as missing tooth, skin problems, diabetes, hyperthyroidism, cleft lip and palate and Down syndrome. It is also worth to mention that 4 taurodonts found in Down syndrome occurred with tooth agenesis.

## Discussion

Taurodontism is an important finding which demands special attention during cavity preparation, root canal therapy as well as tooth extraction.

The present data indicated that the prevalence of taurodontism in our population is 5.5%. Other reported data in Iran show different results as 7.5% prevalence rate in Yazd which is not clearly defined which teeth were included in the study.^19^ To the best of our knowledge, only two studies have evaluated both premolars and molars reporting the prevalence of taurodonts to be 0.3% and 3.9%, respectively.^14 , 2^

**Figure 1:**
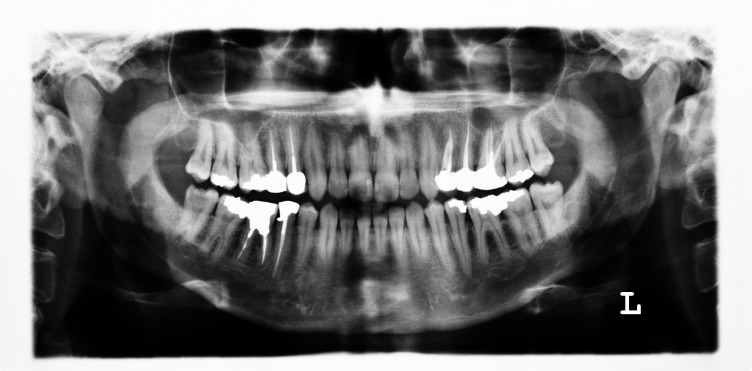
Panoramic view shows bilateral taurodonts in first and second mandibular molars.

The differences between the results of the present study and previous data might have arisen from racial or genetic variations or sample selection and study methods. Including premolars in some studies (as the present one), excluding third molars and reporting prevalence based on the number of affected teeth vs the number of patients are among the most important factors influencing the prevalence rate reported apart from racial differences.

In the present study a higher prevalence of taurodontism was found in females, similar to a study in China, which might be attributed to an overall higher number of females in this study.^2^

Mandibular second molars were the most affected as reported in studies of Americans of European heritage and African Americans.^15 , 20^

No significant differences were found in the type of taurodontism between males and females (P=0.332). Except for the classification used in a study by Keene, no study has ever compared morphologic types for this abnormality.^15^

Taurodontism appears most frequently as an isolated anomaly. However, its association with several syndromes and abnormalities has also been reported.^2^ Many of these disorders have oral manifestations, which can be detected on dental radiographs as alterations in the morphology or chemical composition of teeth; therefore, dentists may be the first to detect them. In this study 10 cases of taurodontism had systemic conditions and 8 had symmetrical taurodonts (Figure 1). Since simultaneous occurrence of anomalies may suggest a genetic predisposition, taurodontism may be associated with several syndromes.^2^

It seems that taurodontism may also provide a valuable clue in detecting probable associated syndromes and other systemic conditions.

## Conclusion

This study showed that the occurrence of taurodontism has a biased racial expression in different populations. Regardless of the prevalence, it is very important for a general dental practitioner to be familiar with taurodontism not only with regards to clinical complications but also due to probable related syndrome and its management.
